# Key players in the regulation of iron homeostasis at the host-pathogen interface

**DOI:** 10.3389/fimmu.2023.1279826

**Published:** 2023-10-24

**Authors:** Inam Ullah, Minglin Lang

**Affiliations:** ^1^ CAS Center for Excellence in Biotic Interactions, College of Life Science, University of Chinese Academy of Sciences, Beijing, China; ^2^ College of Life Science, Agricultural University of Hebei, Baoding, China

**Keywords:** hepatocytes, nutritional immunity, pathogens, siderophores, transferrin

## Abstract

Iron plays a crucial role in the biochemistry and development of nearly all living organisms. Iron starvation of pathogens during infection is a striking feature utilized by a host to quell infection. In mammals and some other animals, iron is essentially obtained from diet and recycled from erythrocytes. Free iron is cytotoxic and is readily available to invading pathogens. During infection, most pathogens utilize host iron for their survival. Therefore, to ensure limited free iron, the host’s natural system denies this metal in a process termed nutritional immunity. In this fierce battle for iron, hosts win over some pathogens, but others have evolved mechanisms to overdrive the host barriers. Production of siderophores, heme iron thievery, and direct binding of transferrin and lactoferrin to bacterial receptors are some of the pathogens’ successful strategies which are highlighted in this review. The intricate interplay between hosts and pathogens in iron alteration systems is crucial for understanding host defense mechanisms and pathogen virulence. This review aims to elucidate the current understanding of host and pathogen iron alteration systems and propose future research directions to enhance our knowledge in this field.

## 1 Introduction

Iron is vital for animals, plants, and microorganisms because of its redox potential. Iron is required as a cofactor for many important biological functions like mitochondrial respiration, oxygen transport, DNA repair and synthesis and the citric acid cycle. Similarly, iron is crucial for many enzymes like cytochromes, aconitases, polymerases, and oxidoreductases (1–3). Therefore, iron is maintained in a critical equilibrium by different protein regulators because both low and high levels of iron can compromise cellular activities. In normal conditions, iron is tightly regulated by absorption, recycling, and intracellular storage (4). Conversely, excessive or unused iron and other transition metals are highly toxic and damaging to cells and to vital organs as they can generate free radicals by the Fenton-Haber Weiss reaction (5, 6). Thereby, iron and other transition metals are very critically monitored to maintain healthy physiological conditions within the body.

High levels of iron have been associated with different pathophysiological conditions like neurodegenerative disorders, cancers, hormonal abnormalities, diabetes, liver and heart disease, and immune system dysfunctions (7, 8). In contrast low levels can lead to iron deficiency, anemia, compromised activation and proliferation of immune cells, and other pathological conditions, so an equilibrium state of iron is mandatory (9, 10). Around 3 to 5 gm of iron is present in normal human adults. In most organisms, the antimicrobial immune pathways and the proliferative capability of many pathogens are limited by the bioavailability of iron (11). When humans and other organisms face pathogens, their bodies’ iron is regulated very strictly by different mechanisms (12, 13) because iron availability is vital for pathogenesis. In most cases the iron level is downregulated by the host to deny available iron to the invading pathogens; in this way, pathogens are iron-starved to death (14). In some cases, the iron level is upregulated aimed to pose the toxic effects of iron to stop the pathogen’s growth (15).

Interestingly, iron also influences the host’s immune response to infection. Iron deficiency has been associated with impaired immune function, making individuals more susceptible to infections. Conversely, excessive iron levels can promote inflammation and contribute to the severity of certain infections, such as malaria (16). The initial importance of host iron withholding during infection comes to the observation in 1946 when the host iron level was significantly declined within 48 hours by *Staphylococcus aureus* inoculation (17). Similarly, iron sequestration and the elevation of different iron regulators have been found during the severe acute respiratory syndrome coronavirus 2 (SARS-CoV-2) infection that causes COVID-19, a globally devastating disease (18). 

**Figure 1:**
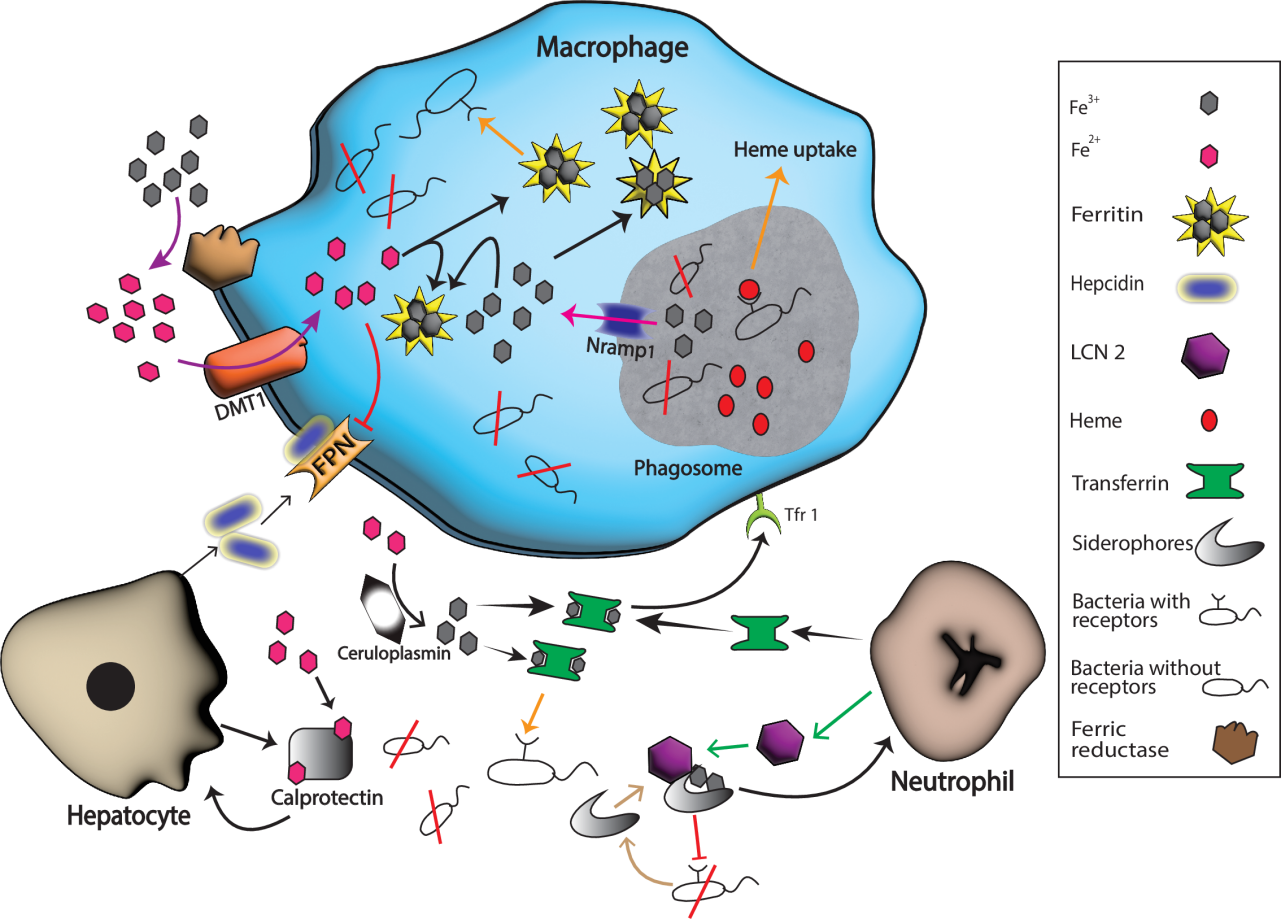
Host and pathogen mechanisms for iron uptake. Hosts produce various substances with a purpose to starve pathogens of iron in response to different inflammatory signals. For example, neutrophil produces transferrin and LCN 2 that binds extracellular Fe^3+^ and bacterial siderophores, respectively while hepatocytes boost the production of calprotectin and hepcidin. Calprotectin binds extracellular Fe^2+^ while hepcidin helps in the degradation of ferroportin 1 (FPN), the known iron exporter, and hence extracellular iron is limited by these actions. Nramp1 production on the surface of phagosomes helps in the export of iron form this compartment, and ferritin stores the intracellular Fe^3+^ that helps in the starvation of intracellular pathogens. Many bacteria escape these barriers because they have specific extracellular receptors for host iron-rich molecules that successfully bind these molecules and fulfill their need for iron for survival (represented by orange arrows). Black arrows represent host iron sequestration mechanisms, while orange arrows represent bacterial iron uptake.

As we know, iron is required for the vital activities of mammals and other organisms (1, 2, 19), therefore, in the majority of vertebrates, iron is stored intracellularly and extracellularly within storage proteins or complexed with cofactors of myoglobin or hemoglobin. In reaction to infectious agents, the host decreases the accessibility of iron in both the intracellular and extracellular spaces by utilizing iron-binding proteins and iron regulatory proteins such as transferrin (Tsf), ferritin, calprotectin, lipocalin 2 (LCN2), etc., to limit the availability of iron to invading pathogens as shown in **Figure 1**. In addition, to make it more difficult for invading pathogens to access host iron, some factors like neutral pH, production of iron-binding proteins and aerobic environment of serum provides additional barriers. Thereby, these numerous barriers make it very difficult for invading pathogens to survive and proliferate (20, 21), And hence, vertebrate hosts have evolved several strategies of iron limitation to prevent bacterial proliferation—a process called nutritional immunity. It is a bacteriostatic strategy of the host immune system that limits many essential metals from pathogens.

However, pathogens have also evolved strategies to overcome a host’s iron restrictions. For instance, certain bacteria produce siderophores which are small molecules that scavenge iron from host proteins and transport it back to the pathogen. This allows the bacteria to acquire iron even in iron-limited environments (22, 23). Once pathogens enter the host, their survival and proliferation depend on the available host iron for completing different synthetic and metabolic reactions (24). Because of the importance of iron to both host and pathogens, there is competition for iron between host and pathogen. Meanwhile, pathogens employ various iron acquisition mechanisms, such as siderophores and specialized receptors, to obtain iron from the host, as shown in **Figure 1** (11, 25, 26). In this review, some of the important impacts of iron regulators that hosts put into action during pathogen attack in an attempt to starve the pathogens to death by sequestration or transport of iron, an important trace element for the survival of pathogens, are highlighted; as are pathogens’ responsive strategies to escape this iron starvation. Understanding the interplay between these key players is crucial for developing strategies to combat iron-related diseases and infections.

## 2 Host iron regulation during pathogen attack

The regulation of iron plays a crucial role in the immune response during a pathogen attack. As we know, iron is an essential nutrient for both host and pathogens, so its availability can impact the outcome of an infection. Pathogens require iron for their growth and survival, while the host employs various strategies to limit its availability to pathogens, thereby restricting their ability to cause infection. During an infection, the host activates a series of defense mechanisms that aim to sequester and limit the access of iron to pathogens as follows;

### 2.1 Transferrin

Transferrins (Tsf) are glycoproteins found in the fluids of vertebrates and invertebrates, having an important role in iron transport and metabolism that bind two iron molecules with high affinity in the extracellular matrix and keep low concentration of iron in blood and other bodily fluids and transport it to various organs (27, 28). Tsf binds approximately all iron released to the plasma from the diet to prevent the formation of free radicals and ensure its transport to target cells (29). It is known that Tsf can inhibit the growth of certain microorganisms due to its strong affinity for iron and hence is involved in the innate immune system of different organisms by sequestering iron from them. There are three homologs of Tsf in insects, i.e. Tsf1, Tsf2, and Tsf3 (30, 31). Different insects like *Drosophila melanogaster* boost the production of Tsf1 when exposed to pathogenic bacteria. Tsf1 is similar to Tsf in mammals (14, 32, 33). However, there is no evidence of the defensive or immune functions of Tsf2 and Tsf3 (31, 34).

Furthermore, Tsf1 transports iron from the gut to the fat body via the hemolymph. The fat body is an important adipose-like tissue in insects that plays a crucial role in various physiological processes, including energy storage, metabolism, and immunity (35, 36). However, the mechanism of relocation of iron from the gut to the fat body needs to be understood. Recently, iron has been detected at high levels in the fat body while at a low level in other tissues during infection, and it is proposed that iron is relocated by Tsf via hemolymph from other tissues toward the fat body in a purpose of limiting the access of the invading pathogens to iron (37). However, the precise mechanism behind the Tsf1 increase cannot be determined, and additional attention is needed in exploring mechanisms contributing to its production and relocation during infection.

In mammals, there are four different types of Tsf, i.e. serum Tsf, lactoferrin (LF), melanotransferrin, and inhibitor of carbonic anhydrase (ICA) (38). Serum Tsf are present in all vertebrates while LF is present in the secretory fluids of mammals. LF can be distinguished from serum Tsf by its different sequence, its higher isoelectric point, and different functions and locations in the body (28). For instance, serum Tsf and LF are two secreted transferrins known to be involved in nutritional immunity by hiding iron to help protect the host from pathogens (39), while the immune functions of melanotransferrin and ICA are not reported. Iron is transported from the blood into the cells by serum Tsf via Tsf receptors. In addition, Tsf receptor expression is downregulated by cytokine signaling in case of intracellular infection in order to stop the import of iron-bound Tsf to cells (40, 41). There is a contrasting regulation of Tsf levels in humans and mice upon infection. Downregulation of Tsf at the site of infection in humans may be due to iron being relocated by serum Tsf to other tissues because many bacterial species can utilize human Tsf for iron acquisition (11, 42). In mice, the disparity in Tsf regulation could be attributed to the distinct pathogen-host interactions and also due to differences in murine protein (43).

**Figure 2:**
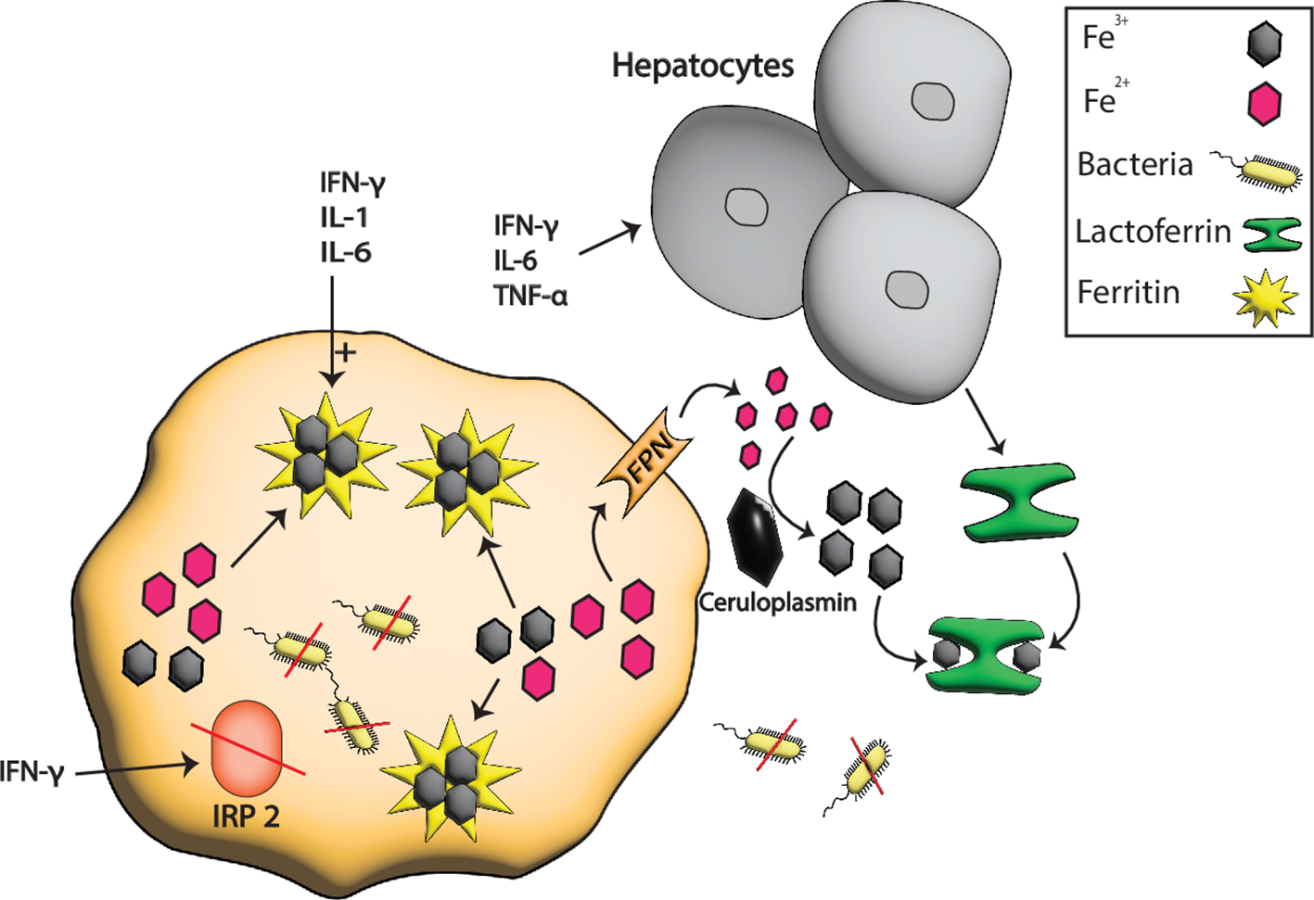
Iron sequestration by LF and ferritin during pathogenesis. At the host-pathogen interface, the hepatocytes boost the production of serum Tsf especially LF in response to different inflammatory signals such as IFN-γ, TNF-α, and IL-6 that sequester the extracellular iron from pathogens. In contrast, the intracellular iron is stored in ferritin whose production is boosted by TNF-α, IL-6, and IL1. IFN-γ also degrades the ferritin repressor IRP2 which further enhances the production of ferritins resulting in more iron storage and hence the starvation of intracellular pathogens due to low or no availability of iron.

Lactoferrin is the major iron-binding protein in human milk and is also found in some specific granules of neutrophils and exocrine gland secretions (44). LF plays an important role in antimicrobial activities as it lowers or stops the proliferative and adhesive capabilities of microbes (45). As LF is present in biological fluids, it lowers the iron concentration in these fluids upon infection and destabilizes the membrane of pathogens. Moreover, in our body, many proteins help regulate iron, but LF is the only regulator that acts as the first line of defense against pathogens entering the body through the mucosa (46, 47). In addition, the hepatic production of LF is increased by IFN-γ, TNF-α, and IL-6 in response to infection that results in the sequestration of extracellular iron, as shown in **Figure 2** (11).

Most of the iron in healthy individuals is intracellular and is bound to the cofactor of cytochromes, ferritin, FeS proteins, and heme erythrocytes (5, 48). After the destruction of erythrocytes, heme and the bounded iron are released into the circulation and are taken up by serum ferroxidase ceruloplasmin (21, 49). LF has the ceruloplasmin binding affinity and once bounded, iron transfer between them is possible. Hence, this transfer prevents the formation of free radicals and the iron acquisition by pathogenic bacteria (50). Moreover, LF can bind iron at acidic pH and can be an effective scavenger of iron in acidotic infection. This can happen in conditions such as diabetic ketoacidosis, sepsis, or certain types of tissue damage. Acidosis not only impairs the immune response but also provides a favorable environment for the growth of iron-dependent microorganisms. Therefore, LF’s ability to sequester iron under acidic conditions is especially important in combating infections in these contexts (51).

Besides the role of LF in host immunity by hiding iron, it also functions in cell differentiation and proliferation. However, the precise mechanisms through which LF influences cell proliferation and differentiation are still being investigated. It is hypothesized that LF may interact with specific cell surface receptors, triggering signaling pathways that regulate gene expression (52). In addition, it is commonly upregulated upon the infection of many microorganisms, so it can be used as a biomarker of infection (53). The iron scavenging activity of LF also has a significant role in different neurodegenerative diseases (5, 54, 55), inflammatory diseases (56), and allergic responses (57). Due to the emergence of multidrug-resistant (MDR) bacterial strains, much research has diverted to using innate molecules as alternatives. Interestingly, LF shows no resistance and has antimicrobial potential against staphylococcus infection and mucormycosis through iron chelation (53, 58, 59), so it can be exploited in combination with other antibiotics as an anti-infective agent.

### 2.2 Ferritin

Ferritins are complex three-dimensional hollow spherical structures that accommodate large quantities of iron in a nontoxic and bioavailable form in its hollow center. They have a mol. wt of about 450 kDa and can store around 4500 ferric iron (Fe^3+^) atoms in their cores. Ferritins are considered as major iron storage protein that is found in plants, animals, and prokaryotes (60, 61). Ferritins, a type of iron-binding protein complex, have been extensively studied for their crucial role in various physiological processes. Recent research has provided evidence to support the significant involvement of higher levels of ferritins in immunity, inflammation, signal transduction, and angiogenesis. By binding and sequestering excess iron, ferritin prevents pathogens from accessing this essential nutrient, limiting their ability to establish an infection. This mechanism is particularly important in the initial stages of infection when the immune system is still mobilizing its defenses (62, 63).

Ferritins are found in both insects and mammals, where they exhibit a common structure consisting of one heavy chain (FTH) and one light chain (FTL). The FTH and FTL serve distinct functions, contributing to the oxidation and nucleation of the iron core, respectively (60, 64). Whereas in plants and bacteria only FTH is present, which helps in the oxidation of only two iron atoms (65). FTH is responsible for detoxification by ferroxidase activity that converts toxic ferrous iron (Fe^2+^) to nontoxic Fe^3+^. Thereby, this activity of FTH helps ferritin sequester iron in its hollow core as hydrous ferric oxide (66). While FTL subunits function in iron nucleation, mineralization, and ferritin protein stability. Together, these chains ensure the proper regulation and storage of iron, safeguarding cellular function and preventing oxidative damage (67). Moreover, FTH expression abnormally increases in different tissues during malignancies and inflammation (68–70).

Ferritin plays an important role in host nutritional immunity, inflammation, and hypoxia (63). In humans and other mammals’ oral cavity, ferritin expression is very high because the oral cavity is exposed to different bacterial species that depend on iron for proliferation, and hence the increased expression of ferritin to sequester iron from bacteria (71, 72). Depending on the context, Ferritin can act as a pro-inflammatory and anti-inflammatory molecule. It can promote inflammation by inducing the production of inflammatory cytokines and chemokines, which attract immune cells to the site of infection. This helps to mount an effective immune response against invading pathogens (73). Conversely, ferritin can also exert anti-inflammatory effects by sequestering iron and preventing its participation in oxidative stress reactions. Iron-mediated oxidative stress can exacerbate inflammation and tissue damage. By binding excess iron, ferritin helps to reduce oxidative stress and limit inflammation, thereby promoting tissue repair and resolution of the immune response (74).

During inflammatory or pathological conditions, ferritin expression is stimulated by pro-inflammatory cytokines such as tumor necrosis factor alpha (TNF-α), interleukin 1 (IL-1), and interleukin 6 (IL-6) through nuclear factor (NF)-κB pathway. In addition, interferon-gamma (IFN-γ) and lipopolysaccharide degrade iron-responsive protein 2 (IRP2), a repressor of ferritin expression, via a nitric-oxide-dependent pathway that initiates the expression of ferritin in macrophages as shown in **Figure 2** (70, 75, 76). Moreover, IL-6 is also an important player in pathological responses because it enhances the expression of FTH and FTL in the hepatocytes (77). All these signaling elevate the level of ferritins that bind and sequester intracellular iron, that lowers the iron level, which is then scarcely available for pathogens.

Ferritin heavy chain is expressed mostly against inflammatory responses while FTL is sensitive to elevated iron levels (78). Ferritins/FTH are also highly expressed in glial cells in mice and *Drosophila* to protect neurons from iron-mediated ferroptotic damage. Ferritin provides an antioxidant defense system to protect neurons from iron-mediated cytotoxicity (79). The main source of ferritin secretion in insects are intestinal cells and is abundant in the fat body, midgut, and hemolyph (37, 80). The synthesis and storage of ferritin differ between mammals and *Drosophila*. In mammals, ferritin primarily originates from hepatocytes or in case of infection from macrophages (60). Recent studies have provided evidence supporting the extraction of small quantities of iron from ferritin by Enterobacteriaceae under conditions of oxidative stress regulation by siderophore-independent mechanisms, enough to promote bacteria survival. However, the exact mechanisms behind this process are still being investigated (81). Therefore, understanding the complex mechanisms by which ferritin participates in these biological processes is crucial for developing targeted therapies and interventions. It was also hypothesized that ferritin can act as an iron exporter and cells can secrete ferritin. The expression of many receptors on the cell surface like Tsf receptor 1, SCARA5, and TIM2 further support this hypothesis. The expression of these receptors are also used as an effective drug delivery system (82, 83).

### 2.3 Hepcidin and ferroportin 1

Hepcidin, initially identified as a cysteine-rich antimicrobial peptide, has been found to play a crucial role in iron regulation during inflammation and infection. It is primarily synthesized in hepatocytes and released from the body through urine, where it was first discovered (84). Hepcidin is also synthesized in other parts, including heart, adipose tissue, kidney, liver, myeloid cells, monocytes, and splenic macrophages (85). This peptide exhibits dual functions, acting as both a potent antimicrobial agent and a key regulator of iron homeostasis. Its significance lies in its ability to modulate iron metabolism in response to various physiological and pathological conditions (86). By inhibiting iron transport across the intestinal epithelium and blocking iron release from macrophages and hepatocytes, hepcidin effectively limits the availability of iron during periods of inflammation and infection. This regulation helps prevent the growth and proliferation of pathogenic microorganisms that depend on iron for survival (87). Hepcidin also plays a crucial role in the pathogenesis of iron-related disorders such as anemia of inflammation and hereditary hemochromatosis (88, 89). Understanding the intricate mechanisms of hepcidin synthesis and its regulatory functions is of great importance in the field of iron metabolism and holds promise for the development of novel therapeutic strategies for various iron-related disorders.

Hepcidin is a very important iron regulator found only in vertebrates. No data related to hepcidin-iron regulation is available in other organisms including insects. However, in insects, divalent metal transporter-1 (DMT1) homolog Malvoli, multicopper oxidase-1, and ferritin have been found to play crucial roles in iron import, export, and storage (90–92). It is reported that *Salmonella typhimurium* infection induces hepcidin formation via the estrogen-related receptor γ (ERR), thereby counteracting nitric oxide (NO) and nuclear factor erythroid 2-related factor-2 (Nrf2) mediated iron export from macrophages exerting antimicrobial effect on *S. typhimurium*. Conversely, it also results in an increased supply of the metal for intracellular microbes (93, 94). Induction of hepcidin also results in the development of hypoferremia and eventually, anemia which are well-known mechanisms occurring upon inflammation and infection (89). Interestingly, a study conducted on fish also reported the induction of hepcidin in response to infection and inflammation. In this particular study, fish were infected with *Streptococcus iniae*, a bacterium known to cause infections in aquatic animals. The researchers found that the level of hepcidin mRNA increased significantly by a staggering 4500 times (95).

**Figure 3:**
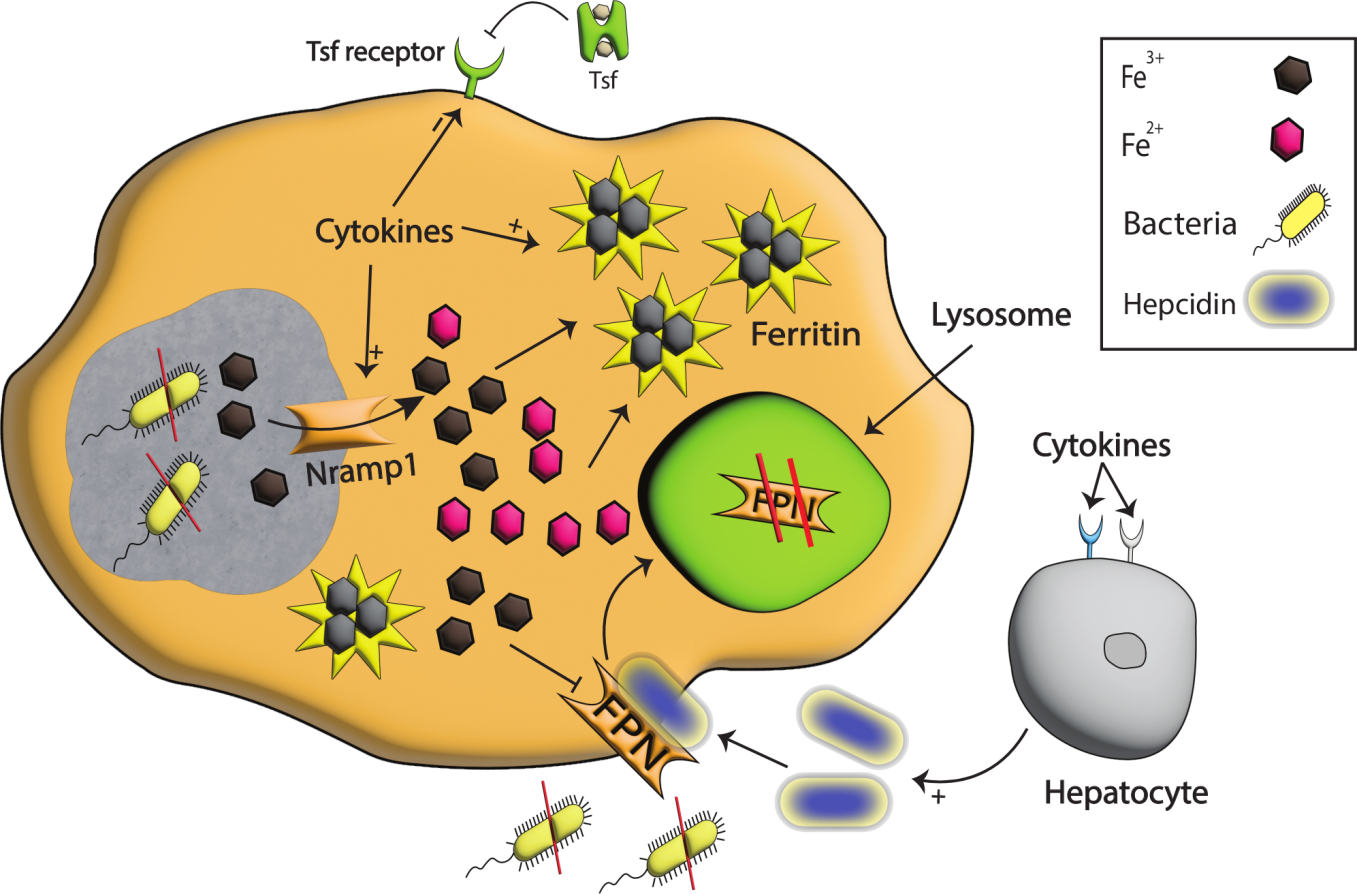
Iron modulation by Nramp1, Hepcidin, and FPN during infection. With the detection of bacterial species, pro-inflammatory cytokines are produced that signal hepcidin production. The binding of hepcidin to FPN results in the degradation of FPN in the lysosomes, and as a consequence iron egress from the cell is reduced, and extracellular pathogens are starved. Pro-inflammatory cytokines also enhance the production of Nramp1, a well-known exporter of iron on phagosomes that helps export iron from phagosomes, stored intracellularly in ferritin, and in this way, intracellular pathogens are starved to death. Moreover, the tsf receptor on the cell surface is reduced, restricting iron import to cells and further limiting intracellular iron availability.

The primary mechanism against infection and inflammation involves the activation of macrophages. With the activation of macrophages, pro-inflammatory cytokines are released, especially IL-1 and IL-6 which induces the production of hepcidin. In addition, myeloid cells can also induce hepcidin production via the activation of TRL4 receptors present on macrophages and neutrophils during inflammation, leading to iron modulation and starvation of infectious agents (96). The increased hepcidin binds FPN, a well-known cellular iron exporter, and is then internalized and degraded by lysosomal machinery (86, 97). With the reduction in FPN due to hepcidin-aided degradation, iron export from cells to plasma reduces, limiting extracellular iron to pathogens, as shown in **Figure 3**. This limited iron export has important implications in the context of pathogen control, as it restricts the availability of extracellular iron to invading pathogens.

### 2.4 Nramp1

The natural resistance-associated macrophage protein (Nramp) family contains evolutionarily conserved divalent metal ion transporters, which play important roles in regulating intracellular divalent ion transport and can be found in animals, plants, and bacteria. In animals, Nramp proteins are present in different tissues and organs. They are particularly abundant in cells of the immune system, where they are involved in the transport of divalent metal ions across cellular membranes. This is important for the proper functioning of immune cells, as divalent metal ions are essential for many immune processes (98–100). Nramp1, a member of the Nramp family, is crucial in protecting against certain intracellular pathogens. This protein is also being significant in regulating iron levels during host-pathogen interactions. Notably, Nramp1’s mRNA expression is significantly upregulated in polymorphonuclear leukocytes and macrophages in humans when exposed to inflammatory signals. This suggests that Nramp1 is actively involved in enhancing innate immunity responses. Its ability to respond to inflammatory signals and its high expression in key immune cells highlights the importance of Nramp1 in the host’s defense against pathogens (101, 102).

In addition, Nramp1 mutation is associated with a significant increase in susceptibility to a range of intracellular pathogens, including *Salmonella*, *Leishmania* spp., and *Mycobacteria*. This heightened susceptibility can be attributed to the loss of function of Nramp1, which leads to the increased availability of iron and other divalent metals to these pathogens (103–105). In human, Nramp1 is required for resistance against intracellular bacteria especially those residing inside phagosomes by restricting the availability of Fe^2+^ and Mn^2+^. For instance, pro-inflammatory cytokines and pattern recognition receptor (PRR) production decrease the production of transferrin receptors on phagocytes which in turn enhance the expression of Nramp1. As a result, iron is exported to the cytoplasm, leading to iron starvation of pathogens in this compartment as shown in **Figure 3** (106). Moreover, macrophages are also responsible for the phagocytosis of red blood cells which contain a high quantity of iron, and this iron is transported from phagosomes by Nramp1 after phagocytosis (107). The expression of Nramp1 also induces lipocalin 2 and nitric oxide production that are linked with iron-mediated immunity (108, 109).

Nramp 2 known as DMT1 helps uptake iron across the brush border membrane of intestinal epithelial cells (110). DMT1 also helps combat pathogens as its expression elevates during viral infection (111). Recent studies have shown the role of DMT1 in the immune response of sea cucumbers. Upon bacterial invasion, sea cucumbers exhibit a significant increase in DMT1 expression. This suggests that DMT1 is crucial in modulating iron metabolism as part of the defense response (112). In addition, there is recent evidence on the role of DMT-1 in bacterial infection. In *Salmoenlla* infection DMT-1 expression controlled iron delivery to intracellular bacteria and induced the siderophore scavenger lipocalin-2 (113). However, the upregulation of DMT1 expression indicates its importance but further studies are required to investigate the precise mechanisms by which DMT1 modulates iron metabolism and its impact on bacterial iron starvation. Such knowledge will not only provide insights into the host defense strategies but may also have implications for the development of novel therapeutic approaches targeting bacterial infections.

In plants, Nramp family proteins are located on the plasma membrane, and they help in uptaking iron from the soil, especially during iron deficiency (99, 114). Plant Nramps exhibit sequence similarity with mouse Nramp1. It has been observed that some of the Nramp genes play a role in plant immunity. In the case of *Arabidopsis thaliana*, the AtNramp3 gene is upregulated in leaves following infection. However, it remains unclear whether AtNramp3 is involved in regulating iron homeostasis during infection. Further research is needed to determine the specific role of AtNramp3 in this context (115). Nramp1 homologs have been identified in many bacterial species like *Escherichia coli* and *Mycobacterium tuberculosis* and have been described as divalent metal iron transporters (116, 117). While malvolio is a Nramp1 homolog in insects and functions in the absorption of dietary iron (118, 119), no data is present about iron regulation by malvolio during infection.

### 2.5 Lipocalin-2

Lipocalin-2 (LCN2) plays a central role in protecting and regulating against intracellular infection by scavenging iron (120). LCN2, also known as neutrophil gelatinase-associated lipocalin (NGAL) or siderocalin, belongs to bacteriostatic factors, produced by neutrophil secondary granules and later also reported to be synthesized by macrophages and epithelial cells (121, 122). It has been found to possess bactericidal, anti-stress, and anti-inflammatory effects, making it a crucial component of the immune response. Its expression is induced by pro-inflammatory cytokine signals, i.e. TNF-α, IFN-γ, IL-1β, IL-17, NF-κB, and JAK-STAT signaling pathways (123, 124).

**Figure 4:**
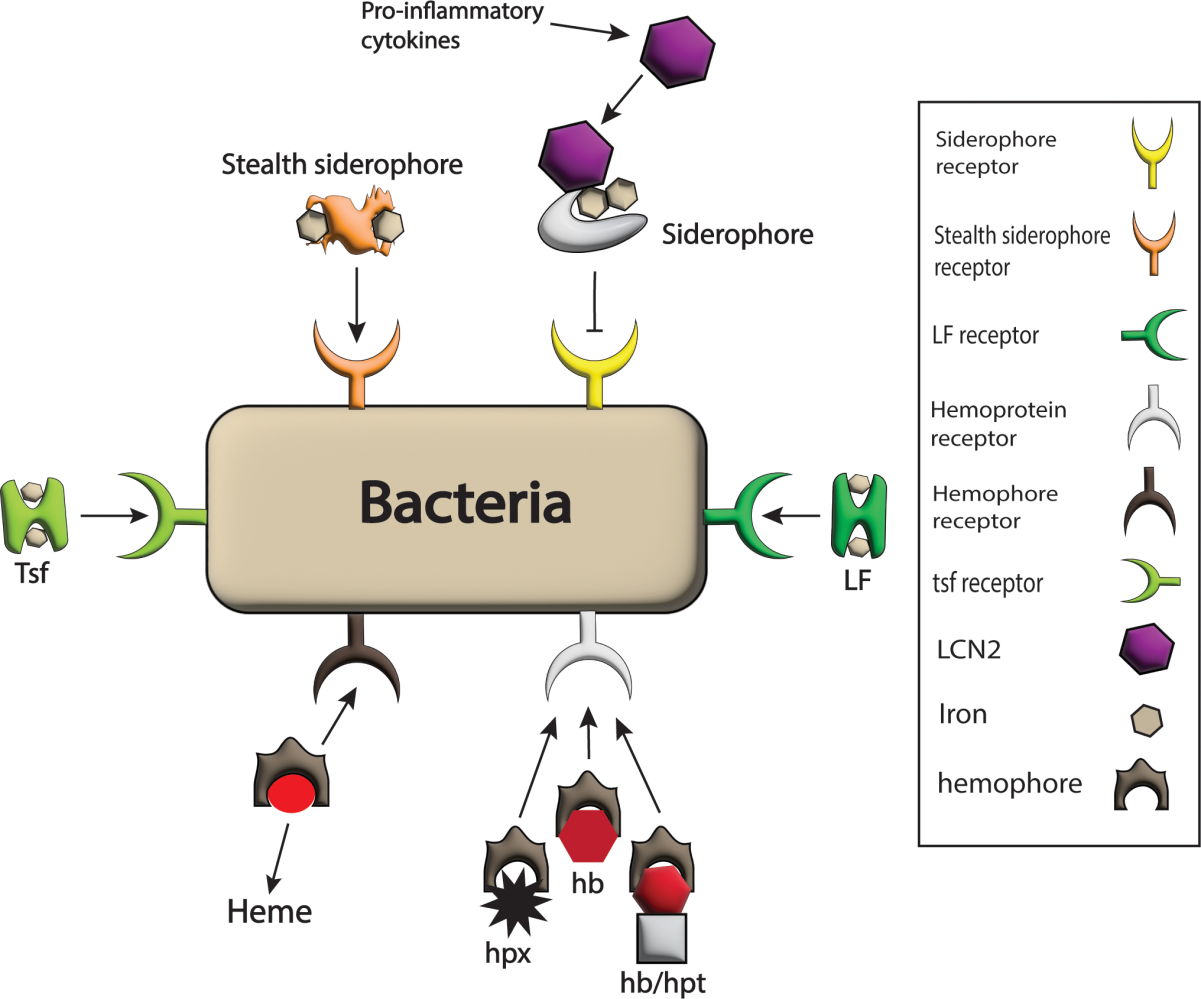
Bacterial strategies against host barriers for iron uptake. The bacterial system expresses different receptors on its surface such as LF receptor, hemophores receptor, Tsf receptor, stealth siderophores receptor, siderophores receptors, and hemoprotein receptor for the acquisition of iron-rich proteins like LF, heme, Tsf, stealth siderophores, siderophores and hpx, hb and hb/hpt complex respectively and hence iron requirements of pathogens are fulfilled. Here only siderophores are blocked from bacteria by the host by the production of LCN2 in response to pro-inflammatory cytokines that bind siderophores.

While LCN2 has various functions, it is best characterized by its ability to inhibit the proliferation of bacteria that rely on siderophores for host iron uptake. Siderophores are specialized proteins produced by bacterial species during infection. These proteins have a high affinity for ferric iron, allowing them to scavenge this essential nutrient from the host (125, 126). However, LCN2 acts as a potent defense mechanism against siderophore-dependent bacteria. It binds to the siderophores, preventing them from delivering iron to the bacterial cells. This effectively limits iron availability for bacterial growth, inhibiting their proliferation and survival within the host, as shown in **Figure 4**. Because of its significant contribution and production, LCN2 has been used as a biomarker in anti-bacterial and anti-inflammatory responses (127, 128).

Accordingly, LCN2 knock-out mice are more susceptible to enterobactin producing bacterial infections (129, 130). Moreover, LCN2 can stop the proliferation of *M. tuberculosis in vitro* and cultured cell lines of macrophages (131, 132), suggesting its role in iron regulation in infection. In animals, one of the crucial molecules 2,5 dihydroxbenzoic acid (2,5-DHBA), which is homologous to siderophores in structure and is being scavenged by some bacterial species, plays an important role in iron homeostasis. During bacterial invasion, this molecule is also downregulated by TLR-mediated pro-inflammatory signaling (133, 134), but the role of 2,5-DHBA downregulation in the innate immune response is still unclear. However, due to the homology of 2,5-DHBA with siderophores, there is a possibility that during bacterial invasion, LCN2 binds 2,5-DHBA to prevent its acquisition by bacteria. Moreover, further research is needed to fully understand the implications of 2,5-DHBA downregulation and the role of LCN2 in the innate immune response. The exact mechanisms by which LCN2 interacts with 2,5-DHBA and its impact on bacterial invasion are still not precise.

### 2.6 Calprotectin

Calprotectin (CT) is a vital component of the innate immune response due to its ability to employ a strategy involving bivalent metal ions withholding. CT is a host-defense bactericidal protein that is primarily expressed by neutrophils and epithelial cells during bacterial invasion, making it an important player in the host defense system (135). CT, previously known for its role in scavenging zinc and manganese during microbial invasion, has recently been found to have a broader function in sequestering Fe^2+^ from extracellular pathogens (136, 137). The role of Fe^2+^ in the host innate immune response has been overlooked, but studies have shown its abundance at the infection site (138). Notably, CT at an infection site poses an innate immune response by taking Fe^2+^, which has recently attracted great attention (139).

Calprotectin is advantageous to the host in response to those pathogens that have the capability of up-taking Fe^2+^ for survival. CT is the only known Fe^2+^ scavenging protein released by neutrophils at the site of infection. CT is also a very critical host metal ions binding protein having two sites for the acquisition of divalent metal ions for antimicrobial effect (139, 140). Some studies have demonstrated that CT can also be used as a biomarker for the diagnosis of different inflammatory and bacterial infections (141–143). It’s proposed that CT and LF/Tsf are part of the first line of host defense against microbial infections and help to limit bacterial multiplication, which is completed at least partly by binding divalent metals like iron to deprive the pathogen (39, 139).

The role of CT in Fe^2+^ sequestration during infection has been questionable for a long time in nutritional immunity, because Fe^2+^ is very unstable under aerobic and oxidative conditions and converts to Fe^3+^ and CT has shown negligible affinity to Fe^3+^. The precise mechanism by which calprotectin withholds Fe^2+^ is still under investigation. However, it is believed that CT binds to free iron ions in the extracellular environment, forming stable complexes that are inaccessible to microorganisms (139, 144, 145). In addition, CT has the capability of maintaining Fe^2+^ oxidation state and can convert Fe^3+^ to Fe^2+^ in aerobic conditions (146). These uncertainties have been tackled in a study conducted on the opportunistic infectious bacteria *Pseudomonas aeruginosa* which has very high Fe^2+^ requirements. This study shows CT-mediated Fe^2+^ sequestration in both aerobic and anaerobic environments that hinder *P. aeruginosa* proliferation (147). Due to the vital role of CT in infection and inflammation by scavenging Fe^2+^, understanding the complex roles of CT and its potential therapeutic applications in the elimination of Fe^2+-^dependent pathogens is crucial in advancing our knowledge in the field of host-pathogen interactions.

### 2.7 Ceruloplasmin

Ceruloplasmin (CP) is a ferroxidase belonging to the multicopper oxidase (MCO) family that stores and carries copper in blood and also functions in iron metabolism, mainly synthesized in hepatocytes and to a lesser extent in lymphocytes and macrophages. CP acts as ferroxidase converting Fe^2+^ to Fe^3+^ which is essential for its incorporation into Tsf, a major iron regulator in nutritional immunity. Both lower and higher levels of CP are linked with different kinds of body disorders (148, 149), and some studies have found upregulation of CP during bacterial invasion (150), suggesting it plays a role not only in iron metabolism but also in iron-mediated nutritional immunity. However, understanding the precise molecular mechanisms by which CP modulates iron-mediated immunity is crucial. This could involve investigating the interaction between CP and immune cells, as well as identifying the signaling pathways involved in ceruloplasmin-mediated immune regulation. Moreover, in insects MCO1 is thought to be involved in gut immunity while it also has been reported to function in the oxidation of Fe^2+^ to Fe^3+^ (92, 151) which may have a close association with nutritional immunity by iron sequestration that needs further investigation.

### 2.8 Haptoglobin and hemopexin

Haptoglobin (hpt) and hemopexin (hpx) are produced in the liver that binds hemoglobin and heme, respectively. These iron-rich proteins are cleared by macrophages from the circulation by phagocytosis, or these complexes are transported to the liver, and the iron is recycled. Hemoglobin and heme in the free state are toxic to tissues;however, they are safe and protected from bacterial uptake when complexed with hpt and hpx (152). While hpt and hpx are primarily known for their involvement in hemoglobin and heme binding, their potential roles in iron transport and distribution within the body warrant investigation. Understanding how these proteins contribute to iron delivery to specific tissues and cells could have implications for the development of targeted therapies for iron-related disorders. Since host iron regulation is very strict and free iron is very roughly available for the invading pathogens, heme is being utilized by some bacterial pathogens to acquire iron (153, 154). Thereby, the expression of hpt and hpx is induced by interleukin-22 (IL-22) at the infectious site, and the induced hpx is recognized to limit heme iron availability to the infectious bacteria *E. coli*. Still, knowledge about hpt iron regulation in this regard is limited (155). However, hpt lowers iron and hemoglobin levels during *S. aureus* septic model induction and that has a significant effect on survival, though *in vivo* studies are limited that can unravel the underlying mechanisms of hemoglobin regulation during pathogen interface (156).

### 2.9 Iron regulatory proteins

Iron regulatory proteins (IRPs) have been explored in the setting of infection. Altered IRP1 and IRP2 expression have been first described upon scrapie infection in the brain of mice (157). Subsequently, Trichomonas vaginalis attracted interest because it can manipulate iron delivery by an iron regulatory binding protein homolog (158). Lastly, IRPs were shown to restrict bacterial iron access and promote iron scavenging via LCN2 in macrophages (159). In iron overload and limited conditions, IRPs detach or bind iron-responsive elements (IRE) on key genes involved in iron metabolism, and thus reduce iron export/import, utilization, and storage according to the underlying pathophysiological conditions or body needs (160, 161). Similarly, IRP1 has been reported to induce the production of Tsf receptors in host cells when challenged with *Toxoplasma gondii*. This indicates that IRP 1 plays a significant role in host innate immunity by modulating iron availability and potentially restricting the parasite’s growth. Further research in this area will help deepen our understanding of the host-parasite interaction and may lead to new interventions against *T. gondii* infection (162).

In short, all the mechanisms at cellular levels are post-transcriptionally under the control of IRP 1 and IRP 2 proteins. In inflammation, infection, iron-limited or excess conditions these proteins help regulate the key genes involved in iron homeostasis. Iron is an essential nutrient for host cells and invading pathogens (163). During infection, hosts put forward different mechanisms to deprive pathogens of iron. But pathogens also have evolved different mechanisms to circumvent the host barriers in order to snatch iron from the host.

## 3 Bacterial iron acquisition against host barriers

Iron is an essential nutrient for the growth and survival of bacteria, but it is often tightly regulated and sequestered by the host as a defense mechanism. In mammals, strict iron regulation leads to minimal levels of free iron in both intracellular and extracellular spaces. Notably, many pathogens have a high dependency on iron for survival and pathogenicity; therefore, they have developed different mechanisms to acquire iron from the host or steal it from host deposition sites (164). Many sites of iron in the host are potentially available for invading pathogens. For instance, pathogens have evolved mechanisms to acquire iron from heme, hpx, hemoglobin, LF, and ferritin, as shown in **Figure 4**.

Many pathogenic microorganisms have evolved the ability to produce siderophores to acquire iron from the host. Different bacteria produce different and very specific siderophores via varying iron binding capacities which are selectively ingested via specific receptors. Siderophores serve as high-affinity iron chelators that scavenge iron from the host’s iron-binding proteins or other sources (165, 166). Special transcription repressors control siderophores expression called ferric uptake regulator (FUR) that binds siderophores genes in iron excess conditions consequently repressing siderophores production. Conversely, under iron-deficient conditions, FUR is inhibited from binding to the DNA binding sites of siderophores genes which results in the expression of siderophores (167, 168). By utilizing siderophores, pathogens gain a competitive advantage in acquiring iron, which is often limited in the host’s environment. This iron piracy mechanism allows them to evade the host’s immune system and proliferate within the host. Siderophores are not only produced by bacteria, but fungi and plants can also produce them during low iron availability (169, 170).

During infection, thehost tightly regulates iron concentrations by producing different iron regulators to prevent its utilization by pathogens (171). Bacteria sense such low iron concentrations, and in response, siderophores are produced to acquire iron with a higher affinity than that of host-produced products. More than 500 siderophores different in structure have been identified. Once siderophores bind Fe^3+^, the complexes are then reclaimed by bacterial cells, where they are docked to the special surface receptors called Fep proteins and internalized for utilization by different mechanisms depending on the type of bacteria (172–174). The siderophores produced by one bacteria might not get back to the same bacteria and hence might not be advantageous to the producer. This may be advantageous to other bacteria of the same species or species with the same siderophores requirements (22). These mechanisms of bacterial siderophore production and acquisition to hijack host iron systems require clearer understanding.

Catecholates, hydroxamates, and carboxylates are the three main siderophore families classified based on structure (175). Conversely, to protect from iron thievery by siderophores, especially catecholates, the host produces LCN2 that can bind and acquire iron from these siderophores (**Figure 4**) (125). However, many bacteria have evolved mechanisms to evade LCN2 inhibition either by expressing stealth siderophores or competitive antagonists that augment the microbial pathogenicity by iron acquisition (166, 169). Additionally, siderophores also scavenge other metals like zinc and copper, protecting some bacteria against oxidative stress (176). Understanding the mechanisms of siderophore production and iron acquisition by pathogens is crucial for developing strategies to disrupt this process and combat infections. Researchers are exploring approaches such as developing siderophore analogs or inhibitors that can disrupt iron uptake and render pathogens more susceptible to host defenses or existing antibiotics. For instance, a novel antibiotic called cefiderocol is already in clinical use, that binds extracellular free iron and is taken up via the siderophore transporters thereby circumvention potential resistance pathways in gram negative bacteria (177). Similarly, by targeting siderophore-mediated iron acquisition pathways, it may be possible to develop novel therapeutic interventions against infectious diseases.

Another mechanism by which some bacteria uptake host iron is by the production of hemophores. Hemophores are specialized proteins that can bind free heme, hpx, hb, and hemoglobin/haptoglobin (hb/hpt) complex. The bacterial cell surface has specialized receptors that can recognize all these substrates, where the complexes are internalized and degraded for the liberation of iron. Heme, being cytotoxic, is accessed by many bacteria by producing exotoxins that can lyse erythrocytes, and the heamoglobin-bound heme is then released (24, 178). In some bacteria heme and heme-associated proteins are directly attached to the bacterial surface, where some specialized proteins internalize them and iron is extracted (164, 179). Though most intracellular and extracellular pathogens utilize the same mechanisms, some intracellular pathogens employ different mechanisms to hijack a host’s iron. For instance, *S. enterica* and *M. tuberculosis* reduce iron export by downregulating FPN expression which allows more intracellular iron for these bacteria (160, 180). In addition, *Francisella tularensis* expresses cell receptors called Tsf receptor 1, FupA, and FslE that successfully acquire iron from Tsf, direct binding of Fe^2+,^and siderophore-mediated Fe^2+^ uptake respectively (181). Another mechanism was observed in a recent study which shows that *Leishmania donovani* can cleave poly(rC)-binding proteins that load iron in ferritin, in macrophages which results in lower loading of iron in ferritin and subsequently its higher availability to the pathogen (182). *Ehrlichia chaffeensis*, the causative agent of human monocytic ehrlichiosis, induces ferritinophagy by producing a protein called Etf-3, which increases the cellular labile iron pool for its proliferation (183).

In addition, some infectious bacterial species have specific membrane receptors, capable of directly binding Tsf and LF which are the key players of nutritional immunity. After binding, iron ions are extracted from Tsf and LF by these special membrane proteins, shifted to periplasm, and then bounded by ferric binding proteins and transported to the cytoplasm where they are utilized by the bacterial cells (42, 184). By employing these strategies, pathogens successfully acquire iron from different sites within the host, ensuring their survival and enhancing their pathogenic potential. Understanding these mechanisms provides valuable insights into the host-pathogen interaction and can open avenues for developing targeted approaches to disrupt pathogen iron acquisition, potentially mitigating infections and related diseases.

## 4 Conclusion

In conclusion, iron plays a multifaceted role in infection. Both bacteria and host have mechanisms to acquire and withhold iron, respectively. In this iron battle, the host efficiently starves and eliminates a number of pathogens; however, some bacteria use more than one system for iron uptake, e.g. the production of different types of siderophores gives some bacteria better chances of survival than others (165). We need a better understanding of how immune cells interact with each other and with pathogens to learn how to modify the iron metabolism in a way that will help us to manipulate host responses to infection and pathogenicity. Moreover, by which pathways host restrict iron availability to extra- and intracellular pathogens are poorly understood. The alteration in either host or pathogen iron sequestration and acquisition pathways pharmacologically may hold key for discovering new approaches that will help prevent or treat infections.

In recent years, significant advancement has been made in developing therapeutic and preventive measures by studying the interconnections between host and pathogens. Taking control over iron is a central part of infection, thus controlling this element could influence the infection in favor of host. For this purpose, siderophores can be modified to be used as iron chelators, or the currently used iron chelators can be improved, aiding the immune system for pathogens’ starvation. Recently, siderophores have been actively exploited as antibiotic carriers to pathogenic bacterial cells, however further investigation is required to enhance the efficacy of this system. Furthermore, all types of siderophores need exploitation to be used as an effective antibiotic-siderophore conjugates delivery system to bacterial cells that will help eliminate a broad range of resistant pathogens. With the increasing knowledge of the metabolic requirements and mechanisms of up taking iron in many pathogens, drug development and modulation of immune pathways are becoming more active research areas that may result in the elimination of antibiotic drug resistance. Future research directions should investigate the role of iron regulatory proteins, host iron sequestration strategies, pathogen iron acquisition systems, and the intricate interplay between host and pathogen in iron alteration processes. By unraveling these mechanisms, we can gain valuable insights into host defense mechanisms and pathogen virulence, ultimately leading to the development of targeted therapeutic approaches.

## Author contributions

ML: Conceptualization, Supervision, Writing – review & editing. IU: Writing – original draft.
